# Cost-effectiveness of procalcitonin testing to guide antibiotic treatment duration in critically ill patients: results from a randomised controlled multicentre trial in the Netherlands

**DOI:** 10.1186/s13054-018-2234-3

**Published:** 2018-11-13

**Authors:** Michelle M. A. Kip, Jos A. van Oers, Arezoo Shajiei, Albertus Beishuizen, A. M. Sofie Berghuis, Armand R. Girbes, Evelien de Jong, Dylan W. de Lange, Maarten W. N. Nijsten, Maarten J. IJzerman, Hendrik Koffijberg, Ron Kusters

**Affiliations:** 1 Department of Health Technology and Services Research, Faculty of Behavioural, Management and Social Sciences, Technical Medical Centre, University of Twente, P.O. Box 217, 7500 AE Enschede, the Netherlands; 2 Department of Intensive Care, Elisabeth-Tweesteden Ziekenhuis, Tilburg, the Netherlands; 3 Department of Critical Care, University Medical Center Groningen, University of Groningen, Groningen, the Netherlands; 4 Department of Intensive Care, VU University Medical Center, Amsterdam, the Netherlands; 5 Department of Intensive Care, Medisch Spectrum Twente, Enschede, the Netherlands; 6 Department of Intensive Care, University Medical Centre Utrecht, University Utrecht, Utrecht, the Netherlands; 7 Laboratory for Clinical Chemistry and Hematology, Jeroen Bosch Ziekenhuis, ‘s-Hertogenbosch, the Netherlands

**Keywords:** Cost-effectiveness, Intensive care, Procalcitonin, Sepsis

## Abstract

**Background:**

Procalcitonin (PCT) testing can help in safely reducing antibiotic treatment duration in intensive care patients with sepsis. However, the cost-effectiveness of such PCT guidance is not yet known.

**Methods:**

A trial-based analysis was performed to estimate the cost-effectiveness of PCT guidance compared with standard of care (without PCT guidance). Patient-level data were used from the SAPS trial in which 1546 patients were randomised. This trial was performed in the Netherlands, which is a country with, on average, low antibiotic use and a short duration of hospital stay. As quality of life among sepsis survivors was not measured during the SAPS, this was derived from a Dutch follow-up study. Outcome measures were (1) incremental direct hospital cost and (2) incremental cost per quality-adjusted life year (QALY) gained from a healthcare perspective over a one-year time horizon. Uncertainty in outcomes was assessed with bootstrapping.

**Results:**

Mean in-hospital costs were €46,081/patient in the PCT group compared with €46,146/patient with standard of care (i.e. − €65 (95% CI − €6314 to €6107); − 0.1%). The duration of the first course of antibiotic treatment was lower in the PCT group with 6.9 vs. 8.2 days (i.e. − 1.2 days (95% CI − 1.9 to − 0.4), − 14.8%). This was accompanied by lower in-hospital mortality of 21.8% vs. 29.8% (absolute decrease 7.9% (95% CI − 13.9% to − 1.8%), relative decrease 26.6%), resulting in an increase in mean QALYs/patient from 0.47 to 0.52 (i.e. + 0.05 (95% CI 0.00 to 0.10); + 10.1%). However, owing to high costs among sepsis survivors, healthcare costs over a one-year time horizon were €73,665/patient in the PCT group compared with €70,961/patient with standard of care (i.e. + €2704 (95% CI − €4495 to €10,005), + 3.8%), resulting in an incremental cost-effectiveness ratio of €57,402/QALY gained. Within this time frame, the probability of PCT guidance being cost-effective was 64% at a willingness-to-pay threshold of €80,000/QALY.

**Conclusions:**

Although the impact of PCT guidance on total healthcare-related costs during the initial hospitalisation episode is likely negligible, the lower in-hospital mortality may lead to a non-significant increase in costs over a one-year time horizon. However, since uncertainty remains, it is recommended to investigate the long-term cost-effectiveness of PCT guidance, from a societal perspective, in different countries and settings.

**Electronic supplementary material:**

The online version of this article (10.1186/s13054-018-2234-3) contains supplementary material, which is available to authorized users.

## Background

Procalcitonin (PCT) is a biomarker that can be used in addition to traditional markers (i.e. C-reactive protein (CRP)) for diagnosing and monitoring patients with bacterial infections [1]. Findings from several randomised controlled trials (RCTs) indicate that the use of a PCT-guided antibiotic treatment algorithm (i.e. PCT guidance) is likely to contribute to a reduction of antibiotic exposure in septic patients in the intensive care unit (ICU) [2–10], without an adverse effect on health outcomes [2, 3, 5–7, 9, 10]. In addition, a recently published Cochrane review concluded that the use of PCT-guided initiation and duration of antibiotic treatment results in a decreased mortality risk, lower antibiotic consumption, and (consequently) a lower risk of antibiotic-related side effects in patients with acute respiratory tract infections [11]. Yet, the considerable cost of PCT testing compared to other laboratory assays (i.e. CRP) remains an important barrier to broader implementation. This barrier may (partly) result from limited insight into the consequences of this PCT algorithm, as both costs and health outcomes that occur along the diagnostic and treatment pathways have not been analysed in depth. Importantly, previous modelling studies into PCT guidance have suggested that it has the potential to save costs [12–14]. However, those studies were all based on a hypothetical patient population instead of real-life patient outcome data, and proved to strongly depend upon the input parameters used. In addition, as results from cost-effectiveness studies may not be readily transferable between countries, it is uncertain to what extent the results from those previous studies can be applied to the Netherlands. In particular, as antibiotic prescription and the duration of hospitalization are relatively low in the Netherlands compared to other developed countries [15, 16], this will limit the potential impact of PCT guidance.

Recently, the Stop Antibiotics on Procalcitonin guidance Study (SAPS) was performed in 1546 patients in 16 hospitals in the Netherlands to investigate the efficacy and safety of PCT guidance in adult ICU patients with sepsis [10]. This trial found that PCT guidance reduces the median duration of antibiotic treatment from 7 to 5 days, and also found - unexpectedly - that this strategy may improve survival. PCT guidance had no impact on ICU or hospital length of stay. This trial provided extensive real-life data suitable for a cost-effectiveness analysis that allows accurate, patient-level estimates of both costs and health outcomes with respect to PCT use in septic ICU patients. Therefore, we used the SAPS data as the input for a trial-based cost-effectiveness analysis in a Dutch setting, with (on average) low antibiotic use and a short duration of hospital stay [15, 16].

## Methods

### Study design and patients

The patient data were derived from the SAPS [10]. This was a prospective, multicentre, randomised, open-label intervention trial among patients admitted to the ICU of 4 university medical centres and 12 teaching hospitals in the Netherlands. The medical ethical committee of the VU University Medical Centre (Amsterdam, the Netherlands) approved this study for all participating centres. ICU patients were eligible for inclusion if they were aged ≥ 18 years and received their first dose of antibiotics for a presumed or proven infection ≤ 24 h before trial inclusion. Patients could participate only once. Written informed consent was obtained from all patients or from their legal representatives. Patients either received treatment according to PCT guidance (PCT group, *n* = 761) or standard of care (standard of care group, *n* = 785), based on random allocation in a 1:1 ratio. A full description of the study design, baseline patient characteristics, and study findings has been published previously [10, 17].

PCT was not measured in the standard of care group. In the PCT group, PCT was measured daily until ICU discharge or until 3 days after systemic antibiotics were stopped, and the results were made available to the attending physician. In addition, a baseline PCT measurement was performed as close to initiation of antibiotics as possible (at least within 24 h), and also made available to the attending physician. The study protocol advised to discontinue antibiotics if the PCT concentration had decreased by 80% or more of its peak value, or when it reached a value of 0.5 μg/L or less. It was left to the discretion of the attending physician whether to adhere to this stopping advice. In the standard of care group, antibiotics were stopped according to local or national guidelines, and this was also left to the discretion of attending physicians. Except for PCT measurements, all monitoring was similar between the PCT and the standard of care group. There was no loss to follow up. All randomised patients were included in this health economic evaluation.

### Outcome measures and model design

A trial-based analysis was performed to estimate the cost-effectiveness of the use of PCT guidance compared with standard of care. The main outcome measures were defined as the impact on the duration of antibiotic treatment, in-hospital mortality, and healthcare costs. These main outcome measures reflect the data recorded (during the SAPS trial) on resource use and patient outcomes, covering the period from sepsis diagnosis and initiation of systemic antibiotic treatment until hospital discharge. In addition, the secondary outcome measure was defined as the incremental cost per quality-adjusted life year (QALY) gained. This outcome measure is referred to as the incremental cost-utility ratio (ICUR). As the SAPS did not collect data on quality of life (i.e. utilities) among sepsis survivors, these were derived from a Dutch follow-up study [18], and combined with one-year mortality rates from the SAPS study. Short-form 36 (SF-36) scores measured at ICU discharge, hospital discharge, and 3 and 6 months after ICU discharge were converted to mean EuroQol-5D (EQ-5D) values. These four utility scores were extrapolated to estimate the utility one year after ICU discharge, by fitting an exponential function over these data using the R software [19]. An overview of the utilities used (and accompanying references) is provided in Additional file 1: Table S1. As these utility values (and costs among sepsis survivors, which are described in the next paragraph) were based on the literature and thus involved additional assumptions, determination of the ICUR was considered an additional scenario.

In addition, as the SAPS did not collect data on productivity losses among patients with sepsis, incorporating societal costs within this analysis would have required making many assumptions, which would have strongly increased the overall uncertainty in the results. However, as current guidelines for health economic evaluations recommend the use of a societal perspective [20], this analysis was performed as a separate scenario. This analysis included the impact of PCT guidance on QALYs and the costs of lost productivity (as described in more detail in “Model inputs – costs”).

#### Model inputs - resource use

Data on the duration of stay on the ICU and the general ward, antibiotic prescriptions (including the type, duration, dose, and mode of administration) and other treatments administered (i.e. mechanical ventilation, dialysis, selective digestive decontamination (SDD), and selective oral decontamination (SOD)), were derived from the SAPS database. Data on mean working hours were derived from Statistics Netherlands for the scenario that incorporates the estimated costs of productivity losses.

Per patient costs of diagnostic testing were calculated based on the frequency and type of blood cultures, other cultures, PCT tests, and (other) routine laboratory tests that were performed (i.e. CRP, bilirubin, creatinine, leukocyte, and thrombocyte testing). The frequency of each, during the first 28 days of hospitalization, was obtained from the SAPS database. A detailed overview of all resource use parameters, references, and assumptions used is provided in Additional file 1.

#### Model inputs – costs

Tariffs for laboratory tests, blood cultures, and other cultures were derived from the Dutch Healthcare Authority (Nederlandse Zorgautoriteit). In the current analysis, a price of €31.71 per PCT measurement was used [21]. Tariffs for hospital stay on the ICU and general ward (which also includes costs of hospital staff, equipment and overheads) and costs of mechanical ventilation and dialysis, were also obtained from the Dutch Healthcare Authority [21, 22]. Costs of antibiotic therapy were calculated by combining data from individual drug administration records from the SAPS database with their accompanying unit costs (as published previously) [10]. All healthcare-related costs up to one year after ICU admission (including the utilisation of hospital care, long-term (home) care, medication, consultations with the general practitioner, and the use of allied health care and mental health care) were derived from literature [23]. To ensure that these costs involved only healthcare costs *after* the initial hospitalization period, the costs after 6 months of sepsis onset were used for the purpose of the current analysis. All costs were converted to 2017 Euros, using Dutch consumer price index levels [24]. Productivity losses were estimated using the friction cost method. Age-dependent Dutch labour participation rates [25, 26] were multiplied with average, gender-specific Dutch wage rage rates [20], over a friction cost period of 85 days in accordance with Dutch guidelines [20]. The recovery period was set at 12 weeks, which is likely a strong underestimation of reality [27]. This analysis also included costs of productivity losses due to premature mortality. A detailed overview of all cost parameters, references, and assumptions used is provided in Additional file 1.

### Analysis

The data were analysed using R (version 3.4.1) [19]. Multiple imputation was used to handle missing values, as this technique is known to yield more valid results than complete case analysis [28]. This multiple imputation step was performed using the package “mice” (version 2.46) and 10 imputation sets [29]. In order to reflect the uncertainty in cost-effectiveness outcomes, the potential variance within the sample data should be considered. In this regard, bootstrapping was used to incorporate this uncertainty and to obtain the accompanying confidence intervals [30]. This bootstrap consisted of 2 stages: (1) random sampling (selection) of individual hospitals from the 16 participating hospitals in the SAPS study [10] and (2) random sampling (selection) of patients from the hospitals selected in step 1. This first step was repeated until the minimum intended sample size of 631 patients [10] was reached in both the PCT and the control group. This two-stage bootstrapping procedure was repeated until 10,000 samples (i.e. replicated databases) had been generated.

Results for both the primary and secondary outcome measure were presented in a cost-effectiveness plane. In addition, to evaluate the probability that the PCT strategy is cost-effective, the cost-utility analysis (the secondary outcome measure) was presented in a cost-effectiveness acceptability curve, using willingness-to-pay (WTP) thresholds ranging from €0/QALY to €200,000/QALY. In the Netherlands, the WTP threshold depends on the disease burden, ranging from €20,000/QALY for low-burden diseases, up to €80,000/QALY for high-burden diseases. Owing to the severity of sepsis, a WTP threshold of €80,000/QALY is most likely applicable, although the results will also be analysed using a WTP threshold of €20,000/QALY. Similarly, the high WTP threshold is also used in interventions for metastatic breast, lung, or colorectal cancer, and for bypass surgery or percutaneous coronary intervention [31–34]. In some conditions, the ICUR may however be much higher, as illustrated by an ICUR of €133,527 per QALY gained for chemotherapy treatment (i.e. cetuximab) vs. best supportive care in metastatic colorectal cancer [34].

As data on costs and utilities were not collected as part of the SAPS (except for costs of antibiotic treatment), these were based on national tariffs and published literature as described previously. To reflect the uncertainty in these mean cost and utility estimates, a scenario was analysed in which the impact of simultaneously varying all cost and utility parameters in the cost-effectiveness analysis was investigated using a normal distribution for costs and a beta distribution for utilities. For costs among sepsis survivors, the standard error was based on published literature [23]. All other parameters were varied assuming a standard error of 10% [35].

## Results

The results of this trial-based cost-effectiveness analysis indicate that the expected in-hospital costs per patient are €46,081/patient in the PCT group, compared with €46,146/patient in the standard of care group. This indicates an average decrease of €65/patient (95% CI − €6314 to €6107, a relative decrease of 0.14%), which can be considered negligible given the degree of uncertainty in this incremental cost estimate. The average in-hospital mortality was 21.8% in the PCT group, compared with 29.8% in the standard of care group, indicating a decrease of 7.9% (95% CI − 13.9% to − 1.8%, i.e. a relative decrease of 26.6%). In addition, the duration of antibiotic use was 6.9 days in the PCT group, compared with 8.2 days with standard of care, i.e. − 1.2 days (95% CI − 1.9 to − 0.4, i.e. − 14.8%). When incorporating healthcare-related costs up to one year after ICU admission, the average costs per patient increased (non-significantly) to €73,665 per patient in the PCT group, compared with €70,961 with standard of care, i.e. + €2704 (95% CI − €4495 to €10,005, i.e. + 3.8%). When considering the impact on QALYs, there was an average of 0.52 QALYs in the PCT group, compared with 0.47 QALYs in the standard of care group, i.e. + 0.05 QALYs (95% CI 0.00 to 0.10, i.e. + 10.1%), resulting in an ICUR of €57,402/QALY gained. Assuming that annually ~ 13,000 adult ICU patients in the Netherlands are diagnosed with sepsis [36], the use of this PCT-guided treatment algorithm could result in an annual increase in total healthcare related costs (up to one year after ICU admission) of approximately €35 M, while saving 612 QALYs.

**Fig. 1:**
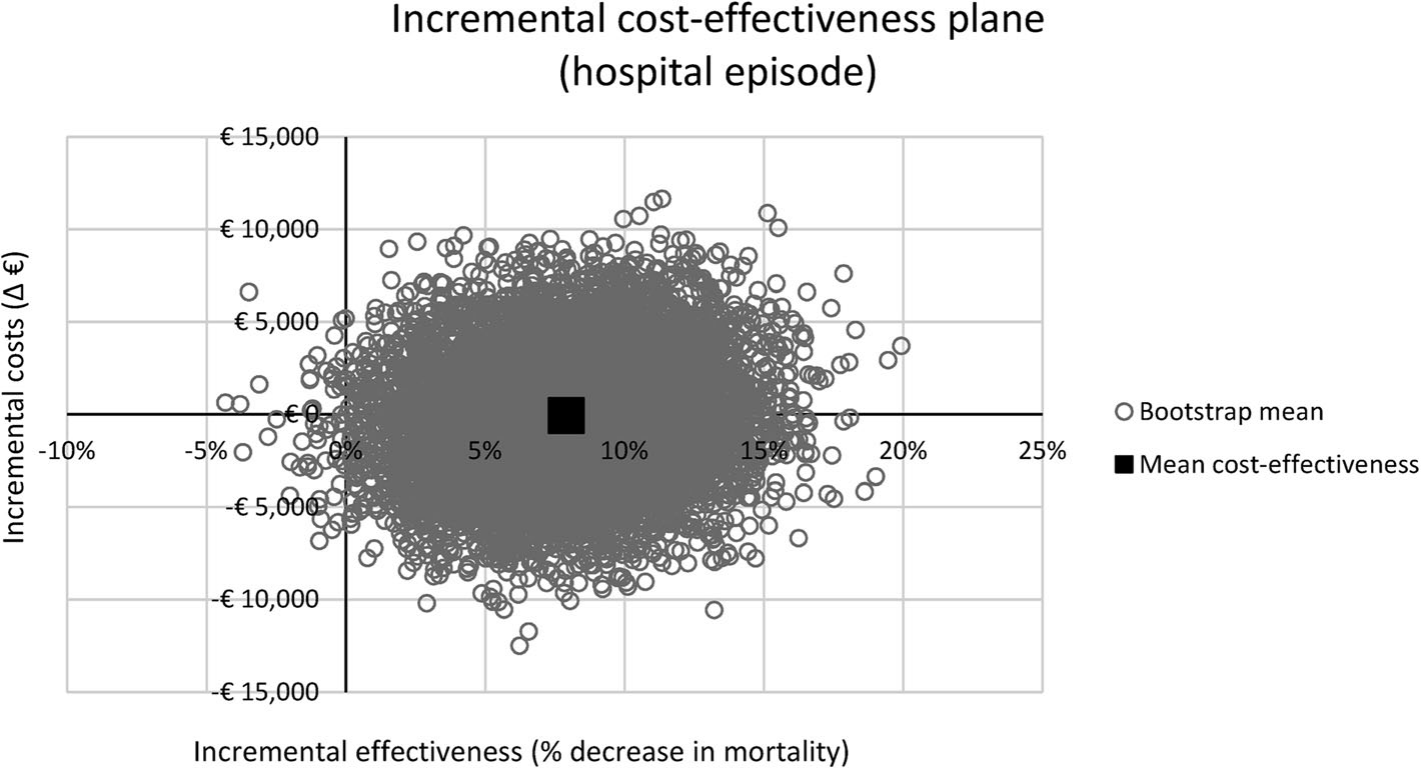
Incremental cost-effectiveness plane for procalcitonin (PCT) guidance compared with standard of care for costs during the hospitalisation episode. This incremental cost-effectiveness plane shows the impact of the use of a PCT-guided antibiotic treatment algorithm, as compared to standard of care, on the difference in in-hospital mortality and accompanying costs within this (initial) hospitalisation episode. The result is based on 10,000 bootstrap samples

**Fig. 2:**
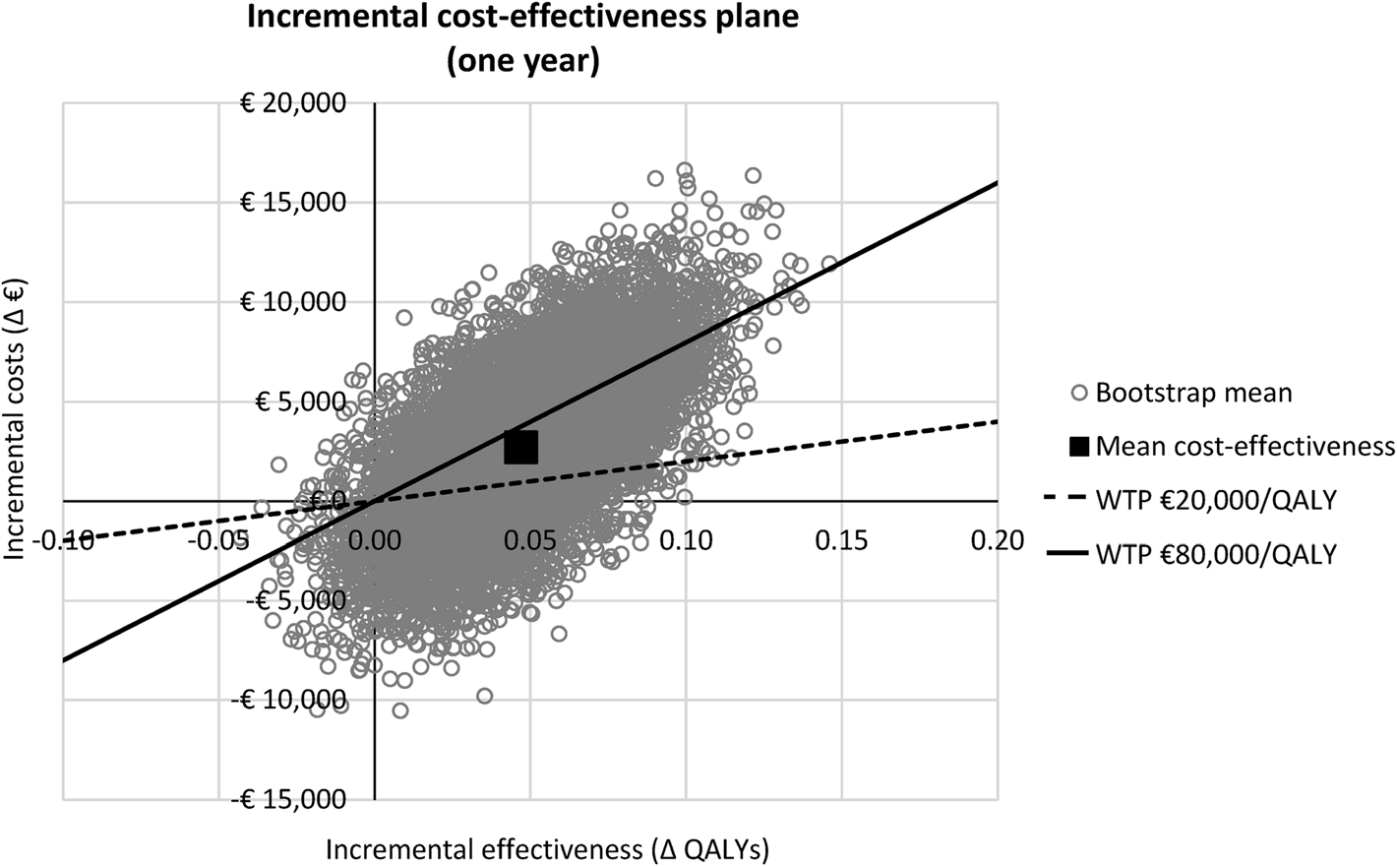
Incremental cost-effectiveness plane for procalcitonin (PCT) guidance compared with standard of care for costs until one year after ICU admission. This incremental cost-effectiveness plane shows the impact of the use of a PCT-guided antibiotic treatment algorithm, as compared to standard of care, on the difference in quality-adjusted life years (QALYs) (until one year after ICU admission) and accompanying healthcare-related costs within this one-year time period. In addition, the willingness-to-pay (WTP) thresholds of €20,000/QALY and €80,000/QALY are shown. The result is based on 10,000 bootstrap samples

A detailed overview of the outcomes of the analyses (including the 95% CIs) is provided in Table 1. The incremental cost-effectiveness plane for the primary outcome measure (i.e. costs per in-hospital death avoided) is shown in Fig. 1 and the incremental cost-effectiveness plane for the secondary outcome measure (i.e. costs/QALY one year after ICU admission) is shown in Fig. 2.

**Table 1:** Overview of model outcomes

Type of parameter	Parameter	PCT, mean (95% CI)	Standard of care, mean (95% CI)	Effect, mean (95% CI)
Effectiveness outcomes				
Hospital stay	ICU stay, days (*n*)	14.6 (12.4 to 16.9)	14.3 (12.5 to 16.1)	0.3 (− 2.2 to 2.8)
	General ward stay, days (*n*)	16.9 (13.9 to 19.6)	17.6 (14.5 to 20.8)	− 0.4 (− 4.3 to 3.5)
Organ support	Mechanical ventilation, days (*n*)	4.7 (4.0 to 5.6)	5.4 (4.5 to 6.3)	− 0.6 (− 1.3 to 0.0)
	Renal replacement therapy, days (*n*)	0.8 (0.5 to 1.1)	0.9 (0.5 to 1.3)	− 0.1 (− 0.5 to 0.2)
Medication	Antibiotics, days (*n*)	6.9 (5.6 to 8.5)	8.2 (7.0 to 9.5)	− 1.2 (− 1.9 to − 0.4)
	SDD and SOD (*n*)	4.0 (0.7 to 8.2)	4.9 (0.9 to 9.9)	− 0.9 (− 2.1 to − 0.1)
Laboratory tests	Cultures (*n*)	4.2 (2.8 to 5.9)	4.8 (3.2 to 6.5)	− 0.5 (− 1.4 to 0.2)
	PCT (*n*)	6.4 (5.7 to 7.3)	0.0 (0.0 to 0.0)	6.4 (5.7 to 7.3)
	Other tests (including order tariff) (*n*)	14.3 (11.3 to 17.3)	14.7 (11.8 to 17.7)	− 0.4 (− 2.8 to 2.1)
In-hospital mortality		21.8% (17.1% to 26.4%)	29.8% (23.5% to 36.4%)	− 7.9% (− 13.9% to − 1.8%)
QALYs		0.52 (0.49 to 0.54)	0.47 (0.43 to 0.51)	0.05 (0.00 to 0.10)
Cost outcomes				
Hospital stay	ICU stay	€32,908 (€28,109 to €38,131)	€32,390 (€28,080 to €36,673)	€519 (− €5227 to €6118)
	General ward stay	€9594 (€6218 to €12,669)	€9972 (€6331 to €13,525)	− €378 (− €2206 to €1300)
Organ support	Mechanical ventilation	€1991 (€1667 to €2369)	€2259 (€1867 to €2671)	− €268 (− €555 to €9)
	Renal replacement therapy	€362 (€244 to €500)	€408 (€247 to €592)	− €46 (− €217 to €116)
Medication	Antibiotics	€203 (€131 to €283)	€237 (€168 to €317)	− €35 (− €73 to €6)
	SDD and SOD	€127 (€32 to €226)	€157 (€42 to €267)	− €30 (− €64 to − €1)
Laboratory tests	Cultures	€109 (€72 to €151)	€122 (€81 to €165)	− €13 (− €34 to €6)
	PCT	€204 (€181 to €232)	€0 (€0 to €0)	€204 (€181 to €232)
	Other tests (including order tariff)	€584 (€484 to €677)	€602 (€502 to €707)	− €19 (− €96 to €56)
Total hospital costs		€46,081 (€38,242 to €54,120)	€46,146 (€39,383 to €53,042)	− €65 (− €6314 to €6107)
Healthcare costs (follow up)		€27,585 (€26,031 to €29,261)	€24,815 (€22,311 to €27,056)	€2770 (€136 to €5550)
Total healthcare costs (up to 1 year follow up)		€73,665 (€66,065 to €81,344)	€70,961 (€64,776 to €77,082)	€2704 (− €4495 to €10,005)
Lost productivity		€6982 (€6582 to €7370)	€6923 (€6570 to €7276)	€59 (− €364 to €485)
Total societal costs (up to 1 year follow up)		€80,647 (€72,918 to €88,401)	€77,884 (€71,604 to €84,116)	€2763 (− €4491 to €10,172)

This table shows an overview of the model outcomes in terms of mean effectiveness and costs on an individual patient level. The mean model outcomes (and accompanying 95% CIs) are shown for the procalcitonin (PCT) group and the standard of care group. In addition, the differences between these groups (and accompanying 95% CIs) are provided

*SDD* selective digestive decontamination, *SOD* selective oral decontamination, *QALY* quality-adjusted life year

Table 1 indicates that when only considering the initial hospitalization episode, the main increase in costs in the PCT compared with the standard of care group is attributable to an increase in the costs of the ICU stay and the costs of PCT testing. The main cost savings are achieved through reduced costs of general ward stay and mechanical ventilation. However, when considering all healthcare-related costs up to one year after ICU admission, an increase in costs of ~ €2700 in the PCT group (compared with standard of care) is expected. In addition, when also including the costs of lost productivity, the difference in costs in the PCT group compared with the standard of care group is expected to increase to ~ €2760. When dividing the incremental costs by the incremental QALYs, results indicate an ICUR of €57,402/QALY gained when considering all healthcare-related costs within one year, and an ICUR of €58,648/QALY gained when also incorporating societal costs.

**Fig. 3:**
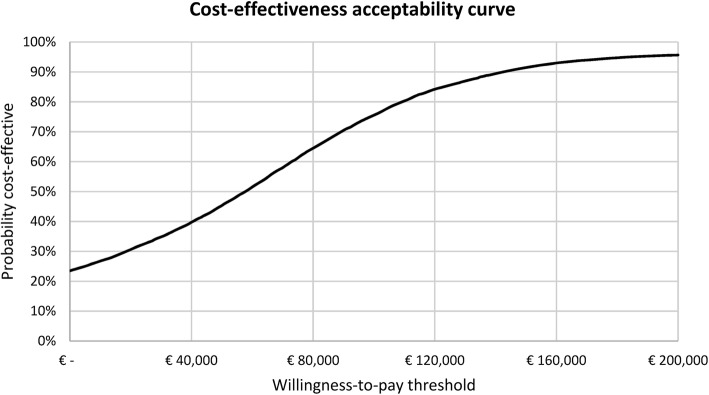
Cost-effectiveness acceptability curve. This cost-effectiveness acceptability curve shows the probability that the use of PCT-guided antibiotic treatment is cost-effective compared to standard of care, for a willingness-to-pay threshold ranging from €0/quality-adjusted life year (QALY) to €200,000/QALY. This analysis incorporates all healthcare-related costs over a one-year time horizon

The results of the cost-effectiveness acceptability curve show the probability that PCT guidance (until one year after ICU discharge) is cost-effective. This probability was found to range from 23.5% to 95.7%, for accompanying WTP thresholds ranging from €0/QALY to €200,000/QALY. For a WTP of €20,000/QALY, this probability was 30.6%, whereas this probability was 64.4% for a WTP of €80,000/QALY (Fig. 3).

In Additional file 2, the model outcomes are shown for the scenario in which all utilities and costs were varied with their (assumed) standard error, including incremental cost-effectiveness planes and the cost-effectiveness acceptability curve. These results indicate that incorporating the uncertainty around utility and cost estimates does increase the width of the 95% CIs in the PCT and the standard of care group, although the 95% CIs of the *incremental* costs are unaffected.

## Discussion

The results indicate that the use of PCT guidance is expected not to affect healthcare costs during the initial hospitalization episode, although there is considerable uncertainty with respect to this incremental cost estimate. This is accompanied by a significant decrease in the duration of antibiotic use and in-hospital mortality. When considering a one-year time horizon, total healthcare-related costs are estimated to increase by 3.8%, which is attributable to high healthcare expenditures in survivors of sepsis. This is accompanied by a (non-significant) 0.05 increase in QALYs, i.e. + 10.1% (until one year after ICU admission), resulting in an ICUR of €57,402/QALY gained. Incorporating the cost of lost productivity will increase the ICUR to €58,648/QALY gained, although uncertainty remains.

### Strengths

The main strength of the current analysis is that the majority of model inputs was derived from a large multicentre RCT. This database had no missing values in the effectiveness variables of interest (i.e. in-hospital mortality and one-year mortality). In addition, the maximum number of missing values for the variables used in the analysis was 5 out of 761 (i.e. 0.7%) and 4 out of 785 (i.e. 0.5%) for “length of ICU stay”, in the PCT and standard of care groups, respectively. Consequently, the impact of multiple imputation on the cost-effectiveness outcomes was negligible, allowing an accurate estimation of the cost-effectiveness of PCT guidance. Furthermore, although (inevitably) some assumptions had to be made, it is very unlikely that these assumptions would have affected the conclusions drawn.

In addition, although the SAPS did not collect data on quality of life (i.e. utilities), or on healthcare-related costs up to one year after ICU discharge, the use of previously published studies performed in the Netherlands [18, 23] has likely contributed to obtaining an accurate estimate of the long-term impact of PCT guidance.

### Limitations

As data on lost productivity were not measured as part of the SAPS, assumptions had to be made to estimate costs of productivity losses due to the recovery period and/or premature mortality. This could be considered as a limitation of the analysis. However, the friction period in the Netherlands is set at 85 calendar days, while the recovery period after hospitalization is set at 84 calendar days, which is likely a strong underestimation [27]. Consequently, every patient will incur the maximum friction period of 85 days regardless of the strategy (i.e. PCT or standard of care) and patient outcomes. The small difference in costs of productivity loss that is observed between the PCT and standard of care strategy, however, is attributable to small differences between the patients included in both groups, in terms of age and gender, resulting in different hourly wage rates.

In addition, as data on resource use on the general ward were not collected as part of the SAPS, we could not account for patient-specific differences in resource use on the general ward, which for example involves differences in undergoing surgical interventions, imaging, blood transfusion, or the use of medication. Therefore, average costs per general-ward day were used instead [37], incorporating costs of medical specialists, nurses, equipment, housing, and overheads. Although this may be considered a limitation, it is expected that the resource use on the general ward will not differ substantially between the PCT and the standard of care group. In addition, costs of resources used during the general ward stay are assumed to be relatively small compared to the high costs during the ICU stay. It is thus highly unlikely that including these costs in more detail would have changed the overall conclusion. Furthermore, as approximately 50% of the infections in the SAPS were hospital or ICU-acquired, it would have been difficult to determine whether these costs on the general ward are indeed related to the sepsis or to the initial reason for hospitalisation.

All model inputs on quality of life were based on a previous study performed by Hofhuis et al. (2008) in the Netherlands [18]. However, this study only concerned patients with *severe *sepsis who were slightly older and who had a longer ICU and hospital length of stay compared to the patients in the SAPS. The quality of life from Hofhuis et al. is therefore likely an underestimation of the utilities in the SAPS. In addition, the main purpose of the current study was to investigate the impact of PCT-guidance on *incremental* QALYs compared to standard of care, which was mainly driven by the difference in mortality rate (as based on the results from the SAPS). Therefore, combining the increased survival in the PCT group with a potential underestimation of the quality of life of these sepsis survivors, likely resulted in a (slight) underestimation of the real-life impact of PCT testing on QALYs, as compared with standard of care.

Results of sensitivity analysis indicate that there is relatively large uncertainty about the impact of PCT guidance on costs (Additional file 2). Although this complicates decision-making, this uncertainty is almost inevitable owing to the fact that sepsis is known to be a highly heterogeneous condition. More specifically, the literature shows that costs rise with increasing sepsis severity [38]. As the SAPS included patients with sepsis, severe sepsis, and septic shock [10], this explains why the duration of ICU and general ward stay varied widely between patients. Consequently, as these parameters are two of the main cost drivers, the uncertainty in total costs remains considerable despite the large number of patients included in the SAPS.

### Recommendations for further research

The adherence to the advice to stop antibiotic treatment (based on the PCT value) was only followed up within 24 h in 44% of the patients [10]. This non-adherence may be explained by reluctance of physicians to stop antibiotic use in patients in an unstable condition. However, over time, physicians may gain trust in PCT testing, which may increase adherence. Consequently, the current study likely underestimated the impact of PCT guidance (in terms of antibiotic use, health outcomes, and costs). Further research is therefore warranted to quantify the full potential impact of this PCT guidance.

Although multiple studies into the impact of PCT guidance have already been published, it was decided not to incorporate these into our analyses as these studies had different goals (i.e. they used a PCT algorithm to decide to start, escalate, and/or discontinue antibiotic treatment [2–10, 39]), and because different patient populations were included (ranging from critically ill adults with undifferentiated infection to surgical ICU patients with severe sepsis) [2–10, 39]). Therefore, performing a meta-analysis was not considered possible. In addition, antibiotic prescription rates and the duration of hospitalisation are relatively low in the Netherlands [15, 16]. Thus, by only using the results of this multicentre trial, it is likely that the benefits shown in this study represent the minimum expected benefits of PCT guidance. The within-hospital benefits of PCT guidance are therefore expected to be larger when performed in other countries, making it also more likely that it may be cost-effective in these countries. However, future studies are recommended to investigate the transferability of the results of the current study to other countries and settings.

Previous research has shown that shorter antibiotic treatment duration likely decreases the incidence of resistant infections and may thereby decrease costs in the PCT group [14]. As the evidence to quantify this impact is limited, this was not incorporated into the current analysis, thereby underestimating the potential further benefits of PCT guidance. Furthermore, the lower mortality rate in the PCT group may incur further benefits to society that cannot be quantified in a health economic evaluation, including the impact on the quality of life of relatives.

Unfortunately, the impact of PCT guidance on long-term survival (i.e. > 1 year) and accompanying long-term costs was not investigated as part of the SAPS and has not been investigated previously [40]. Consequently, the long-term impact of PCT guidance could not be quantified in the current analysis. Furthermore, as the number of studies reporting on long-term survival and healthcare costs among sepsis survivors (in general) is very limited, extrapolating the results of the current study over a longer time period would make the results highly uncertain.

## Conclusions

PCT-guided antibiotic treatment in ICU patients with sepsis is safe and reduces the duration of antibiotic use, while the overall impact on in-hospital costs (i.e. all healthcare-related costs occurring during the initial hospitalisation episode) is negligible (i.e. − €65, − 0.14%). When considering a one-year time frame, the high healthcare-related costs occurring in sepsis survivors may lead to a (non-significant) increase in costs of 3.8% with PCT compared with standard of care. Within this time-frame, the probability of PCT-guidance being cost-effective is 64% at a WTP of €80,000/QALY, although there is substantial uncertainty in the cost estimates. Furthermore, a one-year time frame is too short to capture the full potential impact of PCT guidance. Long-term follow-up studies are required to comprehensively quantify the cost-effectiveness of PCT guidance from a societal perspective. In addition, although it is expected that the benefits of PCT guidance may be more substantial in other countries and settings, this needs to be investigated in future studies.

## Additional files

Additional file 1: Overview of model input parameters and assumptions used. (DOCX 41 kb)

Additional file 2: Results of sensitivity analysis. (DOCX 571 kb)

## Abbreviations

CI: Confidence interval
CRP: C-reactive protein
ICU: Intensive care unit
ICUR: Incremental cost-utility ratio
PCT: Procalcitonin
QALY: Quality-adjusted life year
RCT: Randomised controlled trial
SAPS: Stop Antibiotics on guidance of Procalcitonin Study
SDD: Selective digestive decontamination
SOD: Selective oral decontamination
WTP: Willingness-to-pay
