# Supplementary material for: Cost-effectiveness of procalcitonin testing to guide antibiotic treatment duration in critically ill patients: results from a randomised controlled multicentre trial in the Netherlands
Source: Crit Care. 2018 Nov 13;22:293. doi: 10.1186/s13054-018-2234-3 (PMC6234639; doi:10.1186/s13054-018-2234-3)
Supplement: Supplementary file 1 — Overview of model input parameters and assumptions used. (DOCX 41 kb) [file 13054_2018_2234_MOESM1_ESM.docx]

**Additional file 1: Overview of model input parameters and assumptions used**

***Table S1. Overview of effectiveness input parameters (from literature).*** *This table shows the model input parameters regarding the quality of life among sepsis patients (among patients alive at each time point), and the data source used. ICU = intensive care unit.*

| **Quality of life, at:** | **Value** | **Reference** |
| --- | --- | --- |
| ICU discharge | 0.50 | [1, 2] |
| Hospital discharge | 0.64 | [1, 2] |
| 3 months after ICU discharge | 0.73 | [1, 2] |
| 6 months after ICU discharge | 0.75 | [1, 2] |
| 12 months after study initiation | 0.77 | [1-3] |

***Table S2. Overview of cost input parameters.*** *This table shows the cost input parameters used in the cost-effectiveness analysis, showing the parameter, the value used in the model, and the data source. CI = confidence interval, SDD = selective digestive decontamination, SOD = selective oral decontamination.*

| **Parameter** | **Costs** | **Reference** |
| --- | --- | --- |
| Intensive care unit day   - Category 1 - Category 2 - Category 3 | € 1,854.04  € 2,024.77  € 2,302.53 | [4], assumption  [4], assumption  [4], assumption |
| Mechanical ventilation day   - Category 1 - Category 2 - Category 3 | € 304.07  € 379.93  € 428.51 | [5, 6], assumption  [5, 6], assumption  [5, 6], assumption |
| Regular ward day   - Academic - General | € 447.09  € 647.93 | [6, 7] |
| Dialysis day | € 473.16 | [4] |
| Antibiotic day | Varying (obtained from SAPS) | [8] |
| SOD day | € 1.00 | Elisabeth-TweeSteden Hospital, Tilburg |
| SDD day | € 48.00 | Elisabeth-TweeSteden Hospital, Tilburg |
| Order tariff laboratory tests   - with phlebotomy - without phlebotomy | € 12.03  € 6.27 | [4]  [4] |
| Blood culture | € 31.82 | [4] |
| Other culture | € 19.53 | [4] |
| Determination of positive culture | € 9.89 | [4] |
| Procalcitonin (PCT) | € 31.71 | [4] |
| C-reactive protein (CRP) | € 4.19 | [4] |
| Bilirubin | € 1.54 | [4] |
| Creatinin | € 1.69 | [4] |
| Leukocytes | € 1.85 | [4] |
| Thrombocytes | € 1.83 | [4] |
| Haemoglobin (incl. haematocrit) | € 1.78 | [4] |
| Differential blood cell count | € 1.85 | [4] |
| Glucose | € 1.69 | [4] |
| Potassium | € 1.68 | [4] |
| Sodium | € 1.69 | [4] |
| Urea | € 1.57 | [4] |
| Aspartate aminotransferase (ASAT) | € 1.86 | [4] |
| Alanine-aminotransferase (ALAT) | € 1.96 | [4] |
| Lactate dehydrogenase (LD) | € 1.86 | [4] |
| Gamma-glutamyltransferase(GGT) | €1.84 | [4] |
| Alkaline phosphatase (AF) | € 1.87 | [4] |
| Lactate | € 12.84 | [4] |
| Blood gas | € 5.39 | [4] |
| Urinary sediment | € 2.62 | [4] |
| Productivity losses | Varying (depending on duration of hospital stay and survival) | [9-11] |
| Long-term care costs | Varying (depending on duration of hospital stay and survival) | [8, 12] |

**Assumptions used in the cost-effectiveness analysis:**

- Regarding the costs of an ICU-day:
  - *In the Netherlands, all ICUs have been classified into level 1, 2 and 3, based on ICU size, patient volume, ventilation days, and staffing [13]. This level also determines the tariff that is charged per day that a patient spends on the ICU. Therefore, the costs per ICU day were determined per hospital individually, as based on the classification of the hospitals’ ICUs.*
  - *As this ICU tariff also includes costs of imaging procedures (which were not collected in the database), and which were assumed to be independent of the group the patient is assigned to (e.g. PCT or standard-of-care), costs of these procedures were not separately accounted for in this analysis.*
  - *Costs per day mechanical ventilation were based on the add-on tariff which was used until 2013, which was converted to 2017 Euros. However, as the costs of mechanical ventilation are currently incorporated in the tariff of one day ICU stay, costs of mechanical ventilation were subtracted from this tariff of ICU stay, to avoid double counting of costs. From the SAPS database it was calculated that patients receive, on average, mechanical ventilation for 33% of the time they spend on the ICU.*
- Regarding all laboratory tests:
  - *It is assumed that laboratory tests are performed once-daily until hospital discharge. The order tariff without phlebotomy is used for patients on the ICU (as the blood sample is commonly taken from the central line), while the tariff with phlebotomy is used for patients on the regular ward.*
- Regarding laboratory blood tests:
  - *The number of bilirubin, creatinine, leukocyte, thrombocyte, CRP and PCT tests in the first 28 days of hospitalization was derived from the SAPS database. The assumed set of laboratory tests that was performed upon ICU admission was based on a previous study [14]. For patients hospitalized >28 days, it was assumed that creatinine, leukocyte, thrombocyte and CRP tests were performed once per three days during the hospitalization period.*
  - *Other laboratory tests (haemoglobin, haematocrit, sodium, potassium, urea, and glucose) were assumed to be performed once per three days during the hospitalization period.*
- Regarding blood cultures and other cultures:
  - *During the SAPS study it was recorded how many blood cultures were performed in each patient. In addition, it was also recorded how many other cultures were performed in this study, although it was not specified which type of culture this was. Therefore, we assumed that this concerned a bacteriologic culture, at an average cost of €19.53 [4].*
  - *Both for blood cultures and other cultures, it was assumed that 35% was positive [15] and required determination of the micro-organism.*

**References**

1. Hofhuis, J.G., et al., *The impact of severe sepsis on health-related quality of life: a long-term follow-up study.* Anesth Analg, 2008. **107**(6): p. 1957-64.

2. Ara, R. and J. Brazier, *Deriving an algorithm to convert the eight mean SF-36 dimension scores into a mean EQ-5D preference-based score from published studies (where patient level data are not available).* Value Health, 2008. **11**(7): p. 1131-43.

3. R Core Team, *R: A languange and environment for statistical computing*. 2015, R Foundation for Statistical Computing: Vienna, Austria.

4. Nederlandse Zorgautoriteit, *Tarieventabel_dbc_zorgproducten_en_overige_producten_per_1_januari_2017*. 2017.

5. Nederlandse Zorgautoriteit, *20131010 Tarieventabel DBC zorgproducten en overige producten per 1 oktober 2013.* 2013.

6. Centraal Bureau voor de Statistiek. *Consumentenprijzen; prijsindex 2006 = 100*. 2017 [cited 2017 November 12]; Available from: <http://statline.cbs.nl/StatWeb/publication/?VW=T&DM=SLNL&PA=83131NED>.

7. Hakkaart-van Roijen, L., et al., *Bijlage 1. Kostenhandleiding: methodologie van kostenonderzoek en referentieprijzen voor economische evaluaties in de gezondheidszorg.* 2015, Institute for Medical Technology Assessment, Erasmus Universiteit Rotterdam: Rotterdam. p. 1-120.

8. de Jong, E., et al., *Efficacy and safety of procalcitonin guidance in reducing the duration of antibiotic treatment in critically ill patients: a randomised, controlled, open-label trial.* Lancet Infect Dis, 2016.

9. Zorginstituut Nederland, *Richtlijn voor het uitvoeren van economische evaluaties in de gezondheidszorg*. 2015. p. 1-51.

10. Centraal Bureau voor de Statistiek. *Werkzame beroepsbevolking; arbeidsduur*. 2017 [cited 2017 December 12]; Available from: <https://opendata.cbs.nl/statline/#/CBS/nl/dataset/82647NED/table?ts=1513066092696>.

11. Centraal Bureau voor de Statistiek. *Arbeidsdeelname en werkloosheid per maand*. 2017 [cited 2017 December 12]; Available from: <http://statline.cbs.nl/Statweb/publication/?DM=SLNL&PA=80590ned&D1=8-9&D2=0&D3=0&D4=177-191&VW=T>.

12. Koster-Brouwer, M.E., et al., *Chronic healthcare expenditure in survivors of sepsis in the intensive care unit.* Intensive Care Med, 2016. **42**(10): p. 1641-1642.

13. Kluge, G.H., et al., *The association between ICU level of care and mortality in the Netherlands.* Intensive Care Med, 2015. **41**(2): p. 304-11.

14. Kip, M.M., et al., *A PCT algorithm for discontinuation of antibiotic therapy is a cost-effective way to reduce antibiotic exposure in adult intensive care patients with sepsis.* J Med Econ, 2015. **18**(11): p. 944-53.

15. Singer, M., et al., *The Third International Consensus Definitions for Sepsis and Septic Shock (Sepsis-3).* JAMA, 2016. **315**(8): p. 801-10.
