# Supplementary material for: Cost-effectiveness of procalcitonin testing to guide antibiotic treatment duration in critically ill patients: results from a randomised controlled multicentre trial in the Netherlands
Source: Crit Care. 2018 Nov 13;22:293. doi: 10.1186/s13054-018-2234-3 (PMC6234639; doi:10.1186/s13054-018-2234-3)
Supplement: Supplementary file 2 — Results of sensitivity analysis. (DOCX 571 kb) [file 13054_2018_2234_MOESM2_ESM.docx]

**Additional file 2. Results of sensitivity analysis**

***Table S1. Overview of model outcomes.*** *This table shows an overview of the model outcomes of the sensitivity analysis in terms of effectiveness (i.e. in-hospital mortality, antibiotic days, and QALYs), and in terms of hospital costs. These costs are subdivided into direct hospital costs (consisting of costs of hospital stay, organ support, medication, and laboratory tests), total healthcare-related costs within one year follow-up, and total societal costs which includes productivity losses. The mean model outcomes (and accompanying 95% CIs) are shown for the PCT group and the standard-of-care group. In addition, the difference between these groups (and accompanying 95% CIs) are provided.*

| **Type of parameter** | **Parameter** | **PCT**  **(95% CI)** | **Standard-of-care**  **(95% CI)** | **Effect**  **(95% CI)** |
| --- | --- | --- | --- | --- |
| **In-hospital mortality** | | **21.8%  (17.2% to 26.3%)** | **29.7%  (23.3% to 36.5%)** | **-7.9%  (-13.9% to -1.8%)** |
| **Antibiotic days** | | **6.97  (5.63 to 8.57)** | **8.17  (6.97 to 9.51)** | **-1.20  (-1.93 to -0.39)** |
| **QALYs** | | **0.52  (0.49 to 0.54)** | **0.47  (0.43 to 0.51)** | **0.05  (0.00 to 0.10)** |
| Hospital stay | ICU stay | €32,859  (€25,744 to €41,010) | €32,322  (€25,567 to €39,790) | €537  (-€5,109 to €6,369) |
|  | General ward stay | €9,574  (€6,193 to €13,268) | €9,952  (€6,290 to €14,138) | -€310  (-€662 to €28) |
| Organ support | Mechanical ventilation | €1,986  (€1,532 to €2,544) | €2,252  (€1,735 to €2,859) | -€266  (-€574 to €19) |
|  | Renal replacement therapy | €361  (€229 to €524) | €404  (€233 to €616) | -€44  (-€219 to €116) |
| Medication | Antibiotics | €202  (€131 to €282) | €236  (€168 to €315) | -€34  (-€72 to €6) |
|  | SDD and SOD | €126  (€31 to €231) | €155  (€41 to €274) | -€29  (-€65 to -€1) |
| Laboratory tests | Cultures | €109  (€70 to €156) | €122  (€79 to €170) | -€13  (-€35 to €6) |
|  | PCT | €204  (€159 to €254) | €0  (€0 to €0) | €204  (€159 to €254) |
|  | Other tests (including order tariff) | €582  (€473 to €695) | €601  (€490 to €726) | -€19  (-€96 to €59) |
| ***Total hospital costs*** | | **€46,002  (€36,633 to €56,492)** | **€46,045  (€37,265 to €55,414)** | **-€42  (-€6,279 to €6,443)** |
| Healthcare costs (follow-up) | | €27,606  (€25,415 to €29,797) | €24,855  (€21,997 to €27,574) | €2,751  (€91 to €5,506) |
| **Total healthcare costs (up to one year follow-up)** | | **€73,609  (€64,265 to €84,010)** | **€70,900  (€62,578 to €79,939)** | **€2,709  (-€4,442 to €10,271)** |
| Lost productivity | | €6,981  (€6,591 to €7,367) | €6,924  (€6,566 to €7,270) | €57  (-€359 to €476) |
| **Total societal costs (up to one year follow-up)** | | **€80,589  (€71,137 to €91,013)** | **€77,823  (€69,462 to €86,901)** | **€2,766  (-€4,434 to €10,354)** |

***Figure S1****. Incremental cost-effectiveness plane showing the impact of the use of a PCT-guided antibiotic treatment algorithm, as compared to the standard-of-care, on the difference in in-hospital mortality and accompanying costs within this (initial) hospitalization episode. The result is based on 10,000 bootstrap samples, and based on the scenario in which all utilities and costs were varied with their (assumed) standard error.*

***Figure S2****. Incremental cost-effectiveness plane showing the impact of the use of a PCT-guided antibiotic treatment algorithm, as compared to the standard-of-care, on the difference in QALYs (until one year after ICU admission) and accompanying healthcare-related costs within this one-year time period. In addition, the willingness-to-pay thresholds of €20,000/QALY and €80,000/QALY are shown. The result is based on 10,000 bootstrap samples, and based on the scenario in which all utilities and costs were varied with their (assumed) standard error. QALY = quality-adjusted life year; WTP = willingness-to-pay.*

***Figure S3.*** *Cost-effectiveness acceptability curve showing the probability that the use of a PCT-guided antibiotic treatment is cost-effective compared to the standard-of-care, for a WTP threshold ranging from €0/QALY to €200,000/QALY. This analysis incorporates all healthcare-related costs over a one-year time horizon, and is based on the scenario in which all utilities and costs were varied with their (assumed) standard error. WTP: willingness-to-pay; QALY: quality-adjusted life year.*
